# Human tissue-resident NK cells in the lung have a higher glycolytic capacity than non-tissue-resident NK cells in the lung and blood

**DOI:** 10.1073/pnas.2412489121

**Published:** 2024-10-08

**Authors:** Gráinne Jameson, Aaron Walsh, Robbie Woods, Isabella Batten, Dearbhla M. Murphy, Sarah A. Connolly, Emily Duffin, Oisin O’Gallchobhair, Parthiban Nadarajan, Finbarr O’Connell, Laura E. Gleeson, Joseph Keane, Sharee A. Basdeo

**Affiliations:** ^a^Department of Clinical Medicine, School of Medicine, Trinity Translational Medicine Institute, St James’ Hospital, Trinity College Dublin, Dublin D08 W9RT, Ireland; ^b^Respiratory Department, St James’s Hospital, Dublin D08 NHY1, Ireland

**Keywords:** NK cells, tissue-resident, immunometabolism, lung

## Abstract

Tissue-resident natural killer (trNK) cells are present in the human lung, yet their metabolic function is unknown. NK cell effector and metabolic function are intrinsically linked therefore targeting metabolism presents therapeutic potential in supporting NK cell effector function. This study identifies trNK cells in human bronchoalveolar lavage fluid (BALF) and reveals their distinct metabolic function. To assess the differential phenotype and metabolism of NK cells in the lung, human BALF, and peripheral blood were evaluated by flow cytometry and SCENITH^TM^. Published RNA-sequencing datasets of human lung and blood NK cells were repurposed to determine their differential gene expression. We identified CD49a^+^CD69^+^CD103^+/−^CD56^bright^CD16^−^ trNK cells in human BALF samples and metabolic profiling revealed that lung CD56^bright^CD16^−^ NK cells’ glycolytic capacity and dependence on glucose is significantly higher than matched peripheral blood counterparts. This high glycolytic capacity and glucose dependence was attributed to the trNK cell subset which supports the existing evidence that they have an enhanced ability to respond in the lung.

Natural Killer (NK) cells are innate lymphocytes that have key roles in the early immune response with an ability to be activated without prior sensitization (1). In the blood, they are subdivided into CD56^dim^CD16^+^ and CD56^bright^CD16^−^ subsets which are generally described to be cytotoxic and cytokine producers, respectively. These effector functions are linked to their cellular metabolism with increases in glycolysis and oxidative phosphorylation observed upon activation, both of which are essential for IFN-γ production (2–4). Targeting their cellular metabolism presents extensive therapeutic potential (5); however, the metabolism of distinct tissue-resident NK (trNK) cells in the human lung is unknown (6).

Extensive phenotyping and single-cell RNA-sequencing of human lung tissue biopsies have defined the lung trNK cell subset as CD56^bright^CD16^−^ with coexpression of CD49a, CD69 with or without CD103 (7). Lung trNK cells express higher levels of activating and chemokine receptors and they exhibit a stronger immune response to in vitro influenza infection than non-trNK cells (CD49a^−^CD56^bright^), suggesting an important front-line defense role (8). However, increased frequencies of trNK cells are linked to COPD progression highlighting their potential aberrant responses in disease (9). Understanding the lung trNK cells’ metabolic function will help design metabolism-targeted therapies to modulate NK cell effector function in respiratory diseases.

## Results and Discussion

**Fig. 1.**
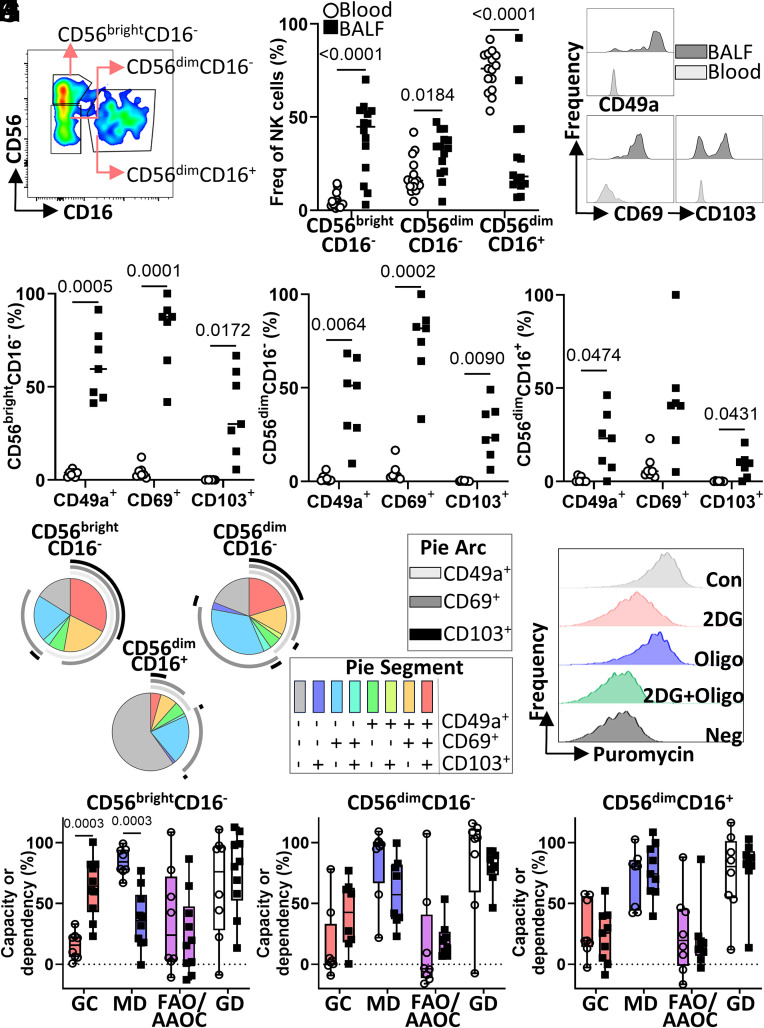
CD56^bright^CD16^−^ NK cells in human BALF have a tissue-resident phenotype and distinct metabolic profile. Mononuclear cells isolated from BALF and peripheral blood were stained with fluorochrome-conjugated antibodies against CD56, CD3, CD69, CD49a, and CD103 and analyzed by flow cytometry or treated with metabolic inhibitors and puromycin for 40 min prior to staining. (*A*) Division of CD56^+^CD3^−^ NK cells. (*B* and *C*) Percentage of CD56^bright^CD16^−^, CD56^dim^CD16^−^, and CD56^dim^CD16^+^ NK cells as a frequency of total live CD56^+^CD3^−^ NK cells in blood (white circle; n = 15) and BALF (black square; n = 14; *B*) and CD49a, CD69, and CD103 expression on CD56^bright^CD16^−^ NK cells (*C*). (*D*–*F*) Percent frequency of CD49a^+^, CD69^+^, CD103^+^ CD56^bright^CD16^−^ NK cells (*D*), CD56^dim^CD16^−^ NK cells (*E*), and CD56^dim^CD16^+^ NK cells (*F*) of total NK cells. (*G*) SPICE pie chart visualizing tissue-residency marker coexpression in BALF (n = 7). (*H*) Representative histogram of puromycin MFI when CD56^bright^CD16^−^cells were treated with control or metabolic inhibitors. (*I*–*K*) Percent glycolytic capacity (GC; red), mitochondrial dependence (MD; blue), fatty acid and amino acid oxidation capacity (FAO/AAOC; purple), and glucose dependence (GD; white) of CD56^bright^CD16^−^ (*I*), CD56^dim^CD16^−^ (*J*), and CD56^dim^CD16^+^ (*K*) NK cells in blood and BALF (n = 8 to 10). Error bars show mean ± SD. P values calculated using two-Way ANOVA and Sidak’s multiple comparison test.

CD49a^+^CD69^+^CD103^+/−^CD56^bright^CD16^−^ NK cells are described as tissue-resident in human lung tissue biopsies (7) and here we confirm their presence in human BALF samples (Patient information in Dataset S1). Access to BALF samples is easier, less invasive, and less time consuming than tissue samples and so, the presence of trNK cells in human BALF samples broadens our research possibilities. We analyzed three NK cell subsets and showed significantly higher frequencies of CD56^bright^CD16^−^ and CD56^dim^CD16^−^ NK cells in BALF compared to peripheral blood (Fig. 1 *A* and *B*). Increased frequencies of CD49a^+^, CD69^+^, and CD103^+^ CD56^bright^CD16^−^ NK cells were observed in BALF compared to blood (Fig. 1 *C* and *D*). Although increased frequencies of CD49a^+^, CD69^+^, and CD103^+^ CD56^dim^CD16^−^ (Fig. 1*E*) and CD56^dim^CD16^+^ (Fig. 1*F*) NK cells were also evident in BALF, SPICE (*SI Appendix*, *Extended Methods*) analyses revealed that the majority lack CD49a and CD103 expression (light blue and gray, Fig. 1*G*). However, analysis of CD56^bright^CD16^−^ NK cells revealed an enrichment of CD49a^+^CD69^+^CD103^+/−^ trNK cells in BALF samples (red and yellow; Fig. 1*G*).

**Fig. 2.**
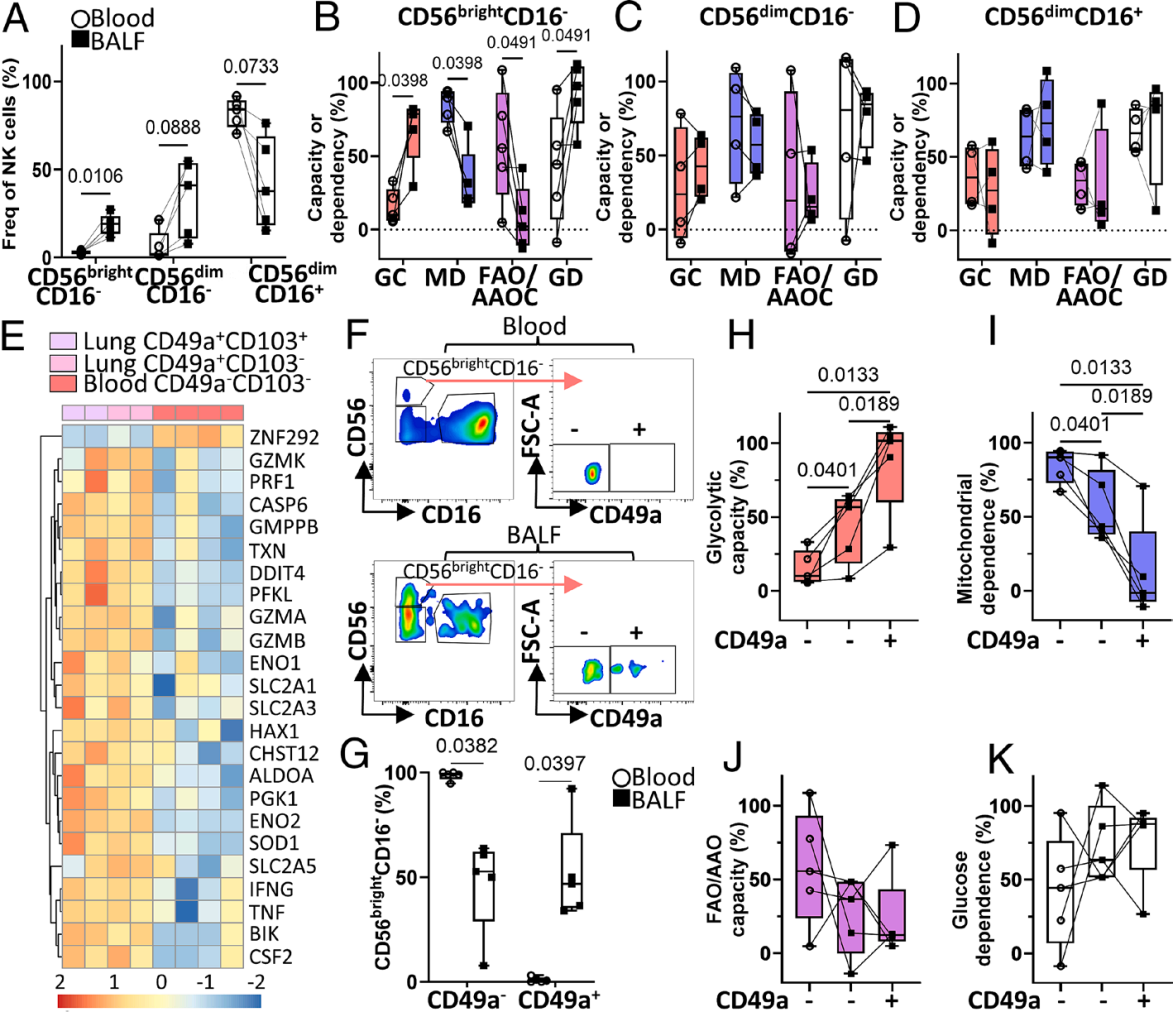
Lung trNK cells have a higher glycolytic capacity than non-trNK in lung and blood. Mononuclear cells isolated from matched BALF and blood were treated with metabolic inhibitors and puromycin for 40 min then stained with fluorochrome-conjugated antibody against puromycin, CD56, CD16, CD3, and CD49a and analyzed by flow cytometry (n = 5). (*A*) Percentage of CD56^bright^CD16^−^, CD56^dim^CD16^−^, and CD56^dim^CD16^+^ NK cells as a frequency of total live CD56^+^CD3^−^ NK cells in matched blood and BALF. (*B*–*D*) Percentage of GC (red), MD (blue), FAO/AAOC (purple), and GD (white) of CD56^bright^CD16^−^ (*B*; n = 5) CD56^dim^CD16^−^ (*C*; n = 4) and CD56^dim^CD16^+^ (*D*; n = 4) NK cells. (*E*) Heatmap showing z-scores of differential metabolism and functional gene expression between lung and blood CD56^bright^CD16^−^ NK cells, repurposed from publicly available datasets (6, 7). (*F* and *G*) Gating strategy (*F*) and frequency plot (*G*) for the analysis of CD49a^+/−^CD56^bright^ NK cells in matched BALF and blood. (*H*–*K*) Percentage GC (*H*), MD (*I*), FAO/AAO (*J*), and GD (*K*) of CD49a^+/−^CD56^bright^CD16^−^ NK cells in blood (white circle) and BALF (black square). *P* values calculated using two-Way ANOVA and Sidak’s multiple comparison test.

Evaluating the metabolic function of human tissue-derived immune cells presents challenges including limited access and low cell number. To overcome these challenges, we utilized SCENITH^TM^ (10) (*SI Appendix*, *Extended Methods*) to identify specific NK cell subsets at a single-cell level (Fig. 1*H*). We show that BALF-derived CD56^bright^CD16^−^ NK cells have a higher glycolytic capacity, and therefore lower mitochondrial dependence, than those in blood (Fig. 1I), whereas no difference was observed for CD56^dim^CD16^−/+^ NK cells (Fig. 1 *J* and *K*). Using matched blood and BALF, we confirmed BALF-derived CD56^bright^CD16^−^ NK cells higher glycolytic capacity (Fig. 2 *A* and *B*; n = 5). Interestingly, BALF-derived CD56^bright^CD16^−^ NK cells had a lower capacity for fatty acid or amino acid oxidation, and therefore a higher glucose dependence, than blood counterparts (Fig. 2*B*). No significant metabolic differences were observed for CD56^dim^CD16^−/+^ NK cells (Fig. 2 *C* and *D*) further supporting that their metabolism does not change between blood and lung compartments.

We hypothesized that this higher glycolytic capacity was attributed to trNK cells and so we reanalyzed published RNA-sequencing datasets (6, 7) (*SI Appendix*, *Extended Methods*) to specifically compare lung trNK cells’ and blood CD56^bright^CD16^−^ NK cells’ expression of glycolysis and effector function-related genes. TrNK cells expressed higher levels of genes associated with glycolysis (ENO1/2, GMPPB, and ALDOA), glucose transport (SLC2A1/3), and effector function (IFNG, TNF, and GZMA,B,K) than blood counterparts (Fig. 2*E*). Next, we assessed the metabolic function of trNK cells, using CD49a expression to differentiate trNK cells from non-trNK cells in BALF (Fig. 2 *F* and *G*). We found that BALF-derived CD49a^+^CD56^bright^CD16^−^ trNK cells’ glycolytic capacity was higher, and mitochondrial dependence lower, than both BALF- and blood-derived CD49a^−^CD56^bright^CD16^−^ NK cells (Fig. 2 *H* and *I*). Interestingly, BALF-derived CD49a^−^CD56^bright^ NK cells’ glycolytic capacity was higher than blood CD49a^−^CD56^bright^ NK cells (Fig. 2*H*), suggesting that CD49a^−^CD56^bright^ NK cells in the lung may represent an intermediate between a blood CD49a^−^CD56^bright^ NK cell and a trNK cell. In support of this, blood NK cells have been shown to induce CD49a expression in response to cytokines (11, 12), and lung-derived CD49a^−^CD56^bright^ NK cells upregulate CD49a expression in vitro (7). This may be a homeostatic mechanism to regulate NK cell effector responses in the lung as collagen IV, a known ligand for CD49a, has been shown to block NK cell effector function (13). No difference was observed between CD49a^+^ and CD49a^−^ CD56^bright^CD16^−^ NK cells’ fuel source in the lung (Fig. 2 *J* and *K*), although the higher glucose dependence observed in total CD56^bright^CD16^−^ NK cells compared to peripheral blood (Fig. 2*B*) suggests that glucose is an important fuel for all CD56^bright^CD16^−^ NK cells in the lung.

Our findings, which reveal trNK cells’ high glycolytic capacity and glucose dependence, are in line with current evidence which demonstrates that trNK cells express high levels of activating receptors and exhibit a heightened response to infection (8). Together, these data indicate that trNK cells have an enhanced ability to respond in the lung when glucose becomes readily available during infections (2–4, 14). This establishment of a solid baseline for lung trNK cell metabolism enables the future investigation of metabolic changes in both infectious and noninfectious respiratory disease contexts and highlights trNK cell metabolism as a tractable target for immunomodulatory therapies.

## Materials and Methods

Our study protocol was approved by the Joint Research Ethics Committee of St. James’s and Tallaght Hospitals and by Trinity College Dublin. Human BALF, retrieved at bronchoscopy, and peripheral blood mononuclear cells were isolated from matched (n = 5) and unmatched anonymized healthy donor blood (n = 20), with informed participant consent, as previously described (15). Cells were stained with fluorochrome-conjugated antibodies specific for cell surface markers and puromycin and acquired by flow-cytometry (*SI Appendix*, *Extended Methods*).

## Supplementary Material

Appendix 01 (PDF)

## Data Availability

Previously published data were used for this work (6, 7). All study data are included in the article and/or *SI Appendix*.
