# Supplementary material for: Human tissue-resident NK cells in the lung have a higher glycolytic capacity than non-tissue-resident NK cells in the lung and blood
Source: Proc Natl Acad Sci U S A. 2024 Oct 8;121(42):e2412489121. doi: 10.1073/pnas.2412489121 (PMC11494342; doi:10.1073/pnas.2412489121)
Supplement: Supplementary file 1 — Appendix 01 (PDF) [file pnas.2412489121.sapp.pdf]

PNAS

Supporting Information for

Human tissue-resident NK cells in the lung have a higher glycolytic capacity than non-tissue-resident NK cells in the lung and blood.

Gráinne Jameson[1], Aaron Walsh[1], Robbie Woods[1], Isabella Batten[1], Dearbhla M. Murphy[1], Sarah A. Connolly[1], Emily Duffin[1], Oisin O'Gallchobhair[1], Parthiban Nadarajan[2], Finbar O'Connell[2], Laura E. Gleeson[1,2], Joseph Keane[1,2], Sharee A. Basdeo[1]

[1]School of Medicine, Trinity Translational Medicine Institute, St James' Hospital, Trinity College Dublin, Dublin, Ireland.

[2]Respiratory Department, St James's Hospital, Dublin 8, Dublin, Ireland.

Corresponding Author: Gráinne Jameson

Email: gjameson@tcd.ie

This PDF file includes:

1. Extended Methods.
2. Dataset S1: Patient information for BALF samples.
3. Dataset S2: List of 52 genes used in bioinformatic analyses of RNA-sequencing datasets.

Extended Methods

Human participants

All BALF donors were patients undergoing clinically indicated bronchoscopy and written informed consent was obtained prior to the procedure. Exclusion criteria included age under 18 years, inability to provide written informed consent or a known (or ensuing) diagnosis of malignancy, sarcoidosis, Tuberculosis, HIV, or Hepatitis C. Patients undergoing biopsy as part of bronchoscopy were also excluded. With these criteria, we aimed to recruit people who were as healthy as possible and undergoing routine bronchoscopy. Please see Dataset S1 for more information. Unmatched peripheral blood samples were obtained from healthy controls.

Flow cytometry

Cells were resuspended in 100µl of FcR block (1µl/test; Miltenyi Biotec) and ZombieNIR stain (Biolegend) or Zombie Aqua (Biolegend) in 1X PBS (Gibco). Anti-CD3 PeDazzle594 (1:200, 17A2), anti-CD56 BV421 (1:200, 5.1H11), anti-CD16 PeCy7 (1:400, 3G8), anti-CD45 APC-Cy7 (1:200, HI30), anti-CD49a FITC (1:100, TS2/7), anti-CD103 APC-Cy7 (1:100, Ber-ACT8), anti-CD69 BV510 (1:100, FN50) were purchased from Biolegend and added to the cells for 10 mins at RT. Cells were washed, fixed and permeabilized with Foxp3 staining kit (eBioscience) according to the manufacturer's instructions. Cells were incubated for 30 mins at 4°C with anti-puromycin (R4743L, 1:100) provided by Dr. Rafael Argüello. Cells were acquired on a FACS Canto (BD Biosciences), BD Fortessa, (BD Biosciences) or an Amnis CellStream (Luminex Corporation, Austin, TX, USA). All data analysis was performed using FlowJo software (BD Biosciences). Gating strategy for analysis of NK cells: Singlets>Live>$CD45^+$>Lymphocytes (FSCloSSClo)>$CD3^-CD56^+$>NK cell subsets division as shown in Figure 1A.

SCENITH (Single Cell mEtabolism by profiling Translation inHibition)

This protocol utilises puromycin as a surrogate for protein synthesis as it is incorporated into nascent proteins. By using specific metabolic inhibitors and measuring puromycin incorporation we can estimate the glycolytic capacity (GC), mitochondrial dependence (MD), fatty acid and amino acid oxidation capacity (FAO/AAOC) and glucose dependence (GD) of cells at the single cell level (Fig. 1G). Both PBMC and BALF-derived cells were resuspended in RPMI + 10% FCS at $2x10^6$ cells/mL in 96 well plates and SCENITH metabolic function profiling was carried out. Cells were treated for 40 mins at 37°C, 5% CO_2 with DMSO Control (Co, 0.02% DMSO), 2-Deoxy-D-Glucose (DG; 100mM; Sigma-Aldrich), Oligomycin (O; 1µM; Sigma-Aldrich), a combination of both drugs (DGO), or with a negative control (Harringtonine; Sigma-Aldrich; 2ug/ml). Puromycin (Puro; 10µg/mL; Sigma-Aldrich) was added for the final 35 mins. Cells were washed in PBS and flow cytometry staining was carried out. The following calculations were used:

Co = GeoMFI of anti-Puro-Fluorochrome upon Control treatment
DG = GeoMFI of anti-Puro-Fluorochrome upon 2-Deoxy-D-Glucose treatment
O = GeoMFI of anti-Puro-Fluorochrome upon Oligomycin A treatment
DGO = GeoMFI of anti-Puro-Fluorochrome upon DG+O treatment

Mitochondrial Dependence (MD)=100(Co-O)/(Co-DGO)
Glycolytic Capacity (GC)= 100-100(Co-O)/(Co-DGO)
Fatty Acid Oxidation and Amino Acid Oxidation Capacity (FAOC)= 100-(100(Co-DG)/(Co-DGO)
Glucose Dependence (GD)= 100(Co-DG)/(Co-DGO)
Therefore, GC is the inverse data of MD (i.e. GC= 100- MD) and FAOC is the inverse data of GD (i.e. FAOC= 100-GD).

BALF samples with sufficient cell numbers underwent metabolic functional profiling using SCENITH (n=10). Samples that did not have sufficient cell yields were phenotyped by flow cytometry as outlined above (n= 19).

SPICE

SPICE (Simplified Presentation Of Incredibly Complex Evaluations) version 6.1 (National Institute of Allergy and Infectious Diseases, National Institutes of Health) is a data mining software that analyses large multicolour flow cytometry datasets and organizes the data graphically. Boolean gates were made of $CD49a^{+/-}CD103^{+/-}CD69^{+/-}$ NK cell subsets using FlowJo software and imported into the SPICE software to generate pie charts in Figure 1G which illustrates the proportion of NK cell subpopulations expressing each tissue residency marker and those that are co-expressing 2 or 3 markers concomitantly.

Bioinformatics

RNA-sequencing data were obtained from the following publications and GEO series: Brownlie et al; GSE166654[6] and Marquardt et al; GSE130379[7]. Gene counts were normalized as copies per million. Student's t-tests were used to identify differences in the expression of 52 genes that are associated with glucose transport, glycolysis, and immune response (Dataset S2), and the resulting p-values were adjusted using the Benjamini-Hochberg method. Data was analysed using R (version 4.3.1). Visualization was done using pheatmap (version 1.0.12).

Statistical Analysis

Statistical analysis was performed using GraphPad Prism version 10. The statistical test used is indicated in each Fig. legend. A P-value of <0.05 was considered statistically significant.

Dataset S1: Patient information for BALF samples.

	**Male (n=9)**	**Female (n=10)**
**Age**	56.11+_9.64	56.63+-10.2
**Smoking status**	Smoker (n=3), Ex-Smoker (n=4), Non-Smoker (n=2)	Smoker (n=5), Ex-Smoker (n=3), Non-Smoker (n=2)
**Indication for bronchoscopy**	Haemoptysis (n=6), Cough (n=1), Lung lesion (n=1), Lung nodule (n=1)	Haemoptysis (n=4), Cough (n=1), Chest discomfort (n=1), Inconclusive COVID-19 swab (n=1), unknown (n=3)
**Co-morbidities**	Type II Diabetes (n=3), Rheumatoid Arthritis (n=1), Multiple Sclerosis (n=1)	Asthma (n=2), COPD (n=1), Tracheobronchopathia osteochondroplastica (n=1)

Dataset S2: List of 52 genes used in bioinformatic analyses of RNA-sequencing datasets.

**Gene**			
SLC2A1	SLC2A14	PGAM2	TXN
SLC2A2	ALDOA	PGAM4	CHST12
SLC2A3	BPGM	PGK1	SOD1
SLC2A4	ENO1	PKLR	GZMA
SLC2A5	ENO2	PKM	GZMB
SLC2A6	GAPDH	TPI1	GZMH
SLC2A7	GPI	ZNF292	GZMK
SLC2A8	HK1	HAX1	GNLY
SLC2A9	HK2	BIK	IFNY
SLC2A10	HKDC1	DDIT4	TNFA
SLC2A11	PFKL	PGK1	CSF2
SLC2A12	PFKM	CASP6	IL10
SLC2A13	PGAM1	GMPPB	PRF1
